# Autism Spectrum Disorders Discourse on Social Media Platforms: A Topic Modeling Study of Reddit Posts

**DOI:** 10.1002/aur.70066

**Published:** 2025-06-05

**Authors:** Seraphina Fong, Alessandro Carollo, Giacomo Vivanti, Daniel S. Messinger, Dagmara Dimitriou, Gianluca Esposito

**Affiliations:** ^1^ Department of Psychology and Cognitive Science University of Trento Rovereto Italy; ^2^ A.J. Drexel Autism Institute Drexel University Philadelphia Pennsylvania USA; ^3^ Department of Psychology, Pediatrics, Electrical & Computer Engineering, and Music Engineering University of Miami Coral Gables Florida USA; ^4^ Sleep Education and Research Laboratory, Psychology and Human Development, UCL Institute of Education London UK; ^5^ Psychology and Human Development Department, UCL, IOE Faculty of Education and Society London UK

**Keywords:** autism, autism spectrum disorders, BERTopic, natural language processing, neurodevelopmental conditions, social media, social networking sites, topic modeling

## Abstract

Social media platforms play a crucial role in shaping public perceptions of neurodevelopmental conditions, such as autism spectrum disorders, by providing spaces for community interaction and content sharing. These platforms hold the potential to foster connections and support among autistic individuals while offering valuable insights into their personal experiences and diverse perspectives. However, knowledge is limited on autism‐related content shared within Reddit, one of the most prominent social media outlets. In this study, we aimed to examine discussions and narratives shared on Reddit about autism, with the dual objectives of identifying the main topics of discussion and exploring the lived experiences of autistic individuals. To achieve this, we utilized state‐of‐the‐art natural language processing techniques to perform a topic modeling analysis on 740,042 autism‐related posts collected from Reddit. Converging themes emerged when comparing the largest and most general subreddit in the dataset (*r/autism*) with 15 additional autism‐related subreddits. The most prominent topics of discussion included challenges in social relationships, behaviors such as stimming, and sensory sensitivities. Additional themes highlighted specific emotional experiences and practical concerns, such as managing a diagnosis, navigating intervention options, and coping with daily life. These findings were organized and discussed in relation to social communication differences and restricted, repetitive behaviors, which are frequently highlighted in discussions about autism. At the same time, we acknowledge the perspective of autistic communities, which view these traits as differences rather than deficits, with many challenges arising from societal expectations and the pressure to mask neurodivergent traits. Together, the results provide a comprehensive overview of the most common topics discussed within autism‐related social media content and offer valuable insights into the lived experiences and motivations for social media engagement among autistic individuals.

**Summary**

- This study analyzed over 740,000 posts on Reddit to explore how autism is discussed and experienced by individuals in online communities.
- Key topics included social challenges, sensory sensitivities, and practical concerns like managing a diagnosis or daily life.
- The findings highlight the diverse perspectives of autistic individuals and emphasize how societal expectations can create challenges, offering valuable insights for fostering understanding and support.

## 1 Introduction

Media outlets, such as social media platforms or TV series, play a key role in shaping public perceptions of and awareness about neurodevelopmental conditions, such as autism spectrum disorders. Fictional portrayals, while sometimes inaccurate or stereotypical, can significantly influence societal attitudes and contribute to misconceptions (Nordahl‐Hansen et al. 2018). Nonetheless, the media can raise awareness and understanding of these conditions through content created or shared by people directly affected by, or closely connected to, neurodevelopmental conditions (e.g., families, health professionals) (Bakombo et al. 2023). In this context, social media platforms provide online spaces for community‐driven input, discourse, and content sharing (Bakombo et al. 2023).

Social media content not only reflects society's current views on neurodevelopmental conditions, but also offers opportunities for individuals with these conditions to connect, interact, or seek support in a safe social environment (Amichai‐Hamburger and Furnham 2007; Kalantari et al. 2023; Mazurek 2013; Zhao et al. 2019). One example of this comes from the autistic community (Hedges et al. 2018; Saha and Agarwal 2016; Wang et al. 2020). Research shows that autistic adults often succeed in forming new friendships on social media platforms and report high satisfaction with their online social lives (Van der Aa et al. 2016). Those who use social media platforms also experience greater happiness, as these platforms strengthen their social connections (Ward et al. 2018). In addition to fostering connections within the autistic community, social media platforms such as Reddit provide a rich source of anonymous opinions and experiences, offering a unique opportunity to gain insight into personal experiences and diverse perspectives surrounding autism. Social media also serves as a platform for the dissemination of misinformation about autism. For example, a recent study examining the informational content of TikTok videos associated with the “#Autism” hashtag found that only 27% of the most viewed videos were accurate, while 41% contained inaccurate information and 32% overgeneralized (Aragon‐Guevara et al. 2023).

However, knowledge is limited on the autism‐related content shared within Reddit, one of the most prominent social media outlets. This study aims to leverage the unique perspectives offered by social media platforms on autism to identify the common discussion topics made within Reddit‐based autistic communities. Reddit is an interactive online social news platform that allows registered users to create or join over 100,000 online communities. These online communities are referred to as “subreddits” and are forums of discussion dedicated to specific topics of interest (Reddit 2024). For instance, the subreddit “*r/Autism_Parenting*” (where “r/” signifies a subreddit) offers a space for Reddit users to discuss various experiences regarding raising autistic children.

The present study is based on state‐of‐the‐art natural language processing (NLP) topic modeling techniques, which permit analyzing large‐scale datasets in a data‐driven manner (Fong et al. 2024a). Previous work has utilized traditional NLP methods such as Linguistic Inquiry and Word Count (LIWC) (Bellon‐Harn et al. 2022) or Latent Dirichlet Allocation (LDA) (Kalantari et al. 2023) for topic modeling. For instance, Kalantari et al. (2023) implemented LDA to identify topics from posts made in the *r/autism* subreddit between 2018 and 2020 (Kalantari et al. 2023). However, their analysis was constrained by a dataset of fewer than 10,000 posts. As done in our previous studies (Fong et al. 2024a, 2024b), this study takes a descriptive approach and uses BERTopic (Grootendorst 2022), a transformer‐based NLP approach, to identify discussion topics from a large public dataset of posts (Watchful1, n.d.) across 16 Reddit subreddits. While LIWC provides a structured dictionary‐based approach and LDA extracts probabilistic topic distributions, BERTopic, in contrast, leverages contextual embeddings and clustering, making it more suitable for identifying nuanced topics in large datasets. Additionally, BERTopic has demonstrated competitive performance in terms of topic coherence and topic diversity when compared to other topic modeling methods (e.g., LDA, NMF, T2V‐MPNET, T2VDoc2Vec, CTM) across various datasets (Grootendorst 2022). Moreover, rather than making predictions or assessing the accuracy of the information, this study aims to systematically describe the key themes emerging in conversations within Reddit‐based autistic communities. Two analyses were conducted: (i) an analysis of 300,557 posts from the main *r/autism* subreddit with posts spanning from 2009 to 2023, and (ii) an analysis of 439,485 posts from 15 autism‐related subreddits *r/AutismInWomen, r/AutismTranslated, r/Autism_Parenting, r/AutisticAdults, r/AutisticCreatives, r/AutisticPeeps, r/AutisticPride, r/AutisticWithADHD, r/Autistic, r/SpicyAutism, r/autismmemes, r/evilautism, r/aspergers, r/aspergirls, r/aspergers_dating*, with posts dated from 2010 to 2023.

By leveraging state‐of‐the‐art NLP techniques and a comprehensive dataset, this study aims to highlight valuable insights into the evolving discourse within autistic communities on Reddit derived from a trained BERTopic model rather than predetermined categories. In particular, the study aims to uncover themes that reflect the experiences and challenges of individuals with autism, thereby enhancing the understanding of their perspectives and needs.

## 2 Materials and Methods

The conduction of this study was approved by the University of Trento Ethical Committee (2024‐40 ESA). The methodology used in this paper is based on the approach implemented in Fong et al. (2024a, 2024b).

### 2.1 Data Collection

This study utilizes data sourced from Reddit. Reddit was chosen as the primary data source for this study for a few reasons. Firstly, since users are not constrained by a limited character count (e.g., 280 characters on Twitter/X), the platform enables users to express their opinions in greater detail and engage in nuanced discussions on a variety of topics. Secondly, the platform provides users with anonymity. This anonymity can encourage users to feel more comfortable to freely express their opinions, attitudes, and experiences (Wanchoo et al. 2023). Lastly, data from Reddit has been shown to be valuable in effectively revealing public opinions on a variety of subject matters in previous research (e.g., Williams et al. 2023; Xu et al. 2024; Yao et al. 2023).

The Reddit data used in this study was obtained from a publicly available dataset compiled by authorized Reddit moderators. The dataset contains posts and comments from the top 40,000 subreddits between June 2005 and December 2023 (Watchful1, n.d.). While previous studies (e.g., Wanchoo et al. 2023; Williams et al. 2023; Xu et al. 2024; Yao et al. 2023) utilized the Pushshift Reddit application programming interface (API) (Baumgartner et al. 2020) to extract Reddit data, changes to Reddit's API in June 2023 restricted Pushshift API access to approved moderators.

All subreddits with the keywords “autism”, “autistic”, or “asperger” in their names were identified from the main dataset. We did not include subreddits named with more general terms (e.g., “neurodiversity”) to ensure data cleanliness in investigating only content related to autism and not other neurodevelopmental conditions. We identified 16 subreddits of interest including: *r/autism, r/AutismInWomen, r/AutismTranslated, r/Autism_Parenting, r/AutisticAdults, r/AutisticCreatives, r/AutisticPeeps, r/AutisticPride, r/AutisticWithADHD, r/Autistic, r/SpicyAutism, r/autismmemes, r/evilautism, r/aspergers, r/aspergirls, and r/aspergers_dating*. The title, main body (“selftext”) of the posts, and the dates when they were published were extracted. Due to computational constraints, posts from *r/autism* were analyzed separately from the other 15 subreddits, as it contained the highest number of posts among the selected subreddits. Prior to data preprocessing, the *r/autism* dataset consisted of *N* = 300,557 posts, while the dataset containing data from the other 15 subreddits consisted of *N* = 439,485 posts.

### 2.2 Data Preprocessing

Posts containing photos (e.g., links to .png files as indicated on Reddit), empty posts (null or containing empty strings), and posts marked as [removed] or [deleted] were excluded to ensure the retention of meaningful text. Emojis were also removed, as they primarily convey affect rather than contribute to the thematic structure of discussions, and their meaning is often context‐dependent. Since this study focused on topic modeling rather than sentiment analysis, including emojis could have introduced noise rather than enhanced topic coherence. Removing them ensured that the analysis remained centered on textual content. Additionally, the titles and main bodies of the posts were combined, as titles often provided supplementary informative content.

All remaining posts were converted to lowercase. No additional preprocessing steps, such as tokenization or stop word removal, were taken to maintain the original structure of each post. This allows the transformer‐based topic models to capture the contextual relationships among words within sentences, leading to more accurate numerical representations of the text posts (i.e., embeddings) (Grootendorst, n.d.‐c; Yao et al. 2023).

After preprocessing, the *r/autism* dataset contained *n* = 174,102 posts (from November 2009 to December 2023), while the dataset comprising the remaining 15 subreddits contained *n* = 291,756 posts (from May 2010 to December 2023).

### 2.3 Topic Modeling

This study utilizes a NLP technique called topic modeling to identify the main topics discussed in autism‐related subreddits. This approach implements statistical modeling to extract patterns within a corpus of unstructured text data and extrapolate topics (Egger and Yu 2022). More specifically, the present work uses a topic modeling procedure called BERTopic (Grootendorst 2022). This is because prior research has demonstrated BERTopic's ability to generate interpretable topics from unlabeled Reddit posts (e.g., Williams et al. 2023; Yao et al. 2023).

BERTopic utilizes embeddings generated from Bidirectional Encoder Representations from Transformers (BERT) (Devlin 2018) and class‐based term frequency‐inverse document frequency (c‐TF‐IDF) to categorize posts into semantically related topics (Ng et al. 2023). The BERT embeddings enable BERTopic to create meaningful topic representations by taking word context and word meanings into account (Ng et al. 2023; Grootendorst 2022). On the other hand, c‐TF‐IDF assesses the significance of a word within a group of posts and computes its frequency to generate the topics (Yao et al. 2023).

Two separate BERTopic models were trained: one on the *r/autism* dataset and the other on the dataset containing posts from the remaining 15 selected subreddits. Python version 3.10.12 was utilized for preprocessing and training both BERTopic models. The models were trained using the BERTopic Python library (version 0.16.0) (Grootendorst 2022). An overview of the model hyperparameters can be found in Table S1 of the supplementary materials. While most default settings were preserved, we specified a random state of 42 for the Uniform Manifold Approximation and Projection (UMAP) model to ensure reproducibility as it prevents stochastic behavior (i.e., producing different results each time the model is run). Additionally, extra stop words, such as “http”, “https”, “amp”, and “com” were removed to reduce noise in the generated topics (Grootendorst, n.d.‐b).

### 2.4 Topic Labeling

BERTopic derives topics from each dataset and represents them with relevant keywords and representative posts. By default, the extracted topics are assigned labels from Topic 0 to *N* (where *N* equals the total number of generated topics minus one). To create more informative topic labels, the set of keywords and samples of representative posts were provided to ChatGPT‐4o using the following prompt (Grootendorst, n.d.‐a):

“I have a topic that contains the following documents:

[DOCUMENTS]

The topic is described by the following keywords:

[KEYWORDS]

Based on the information above, extract a short topic label in the following format: topic:

*<*topic label*>*”

## 3 Results

### 3.1 Main Topics of Discussion in r/Autism

**FIGURE 1:**
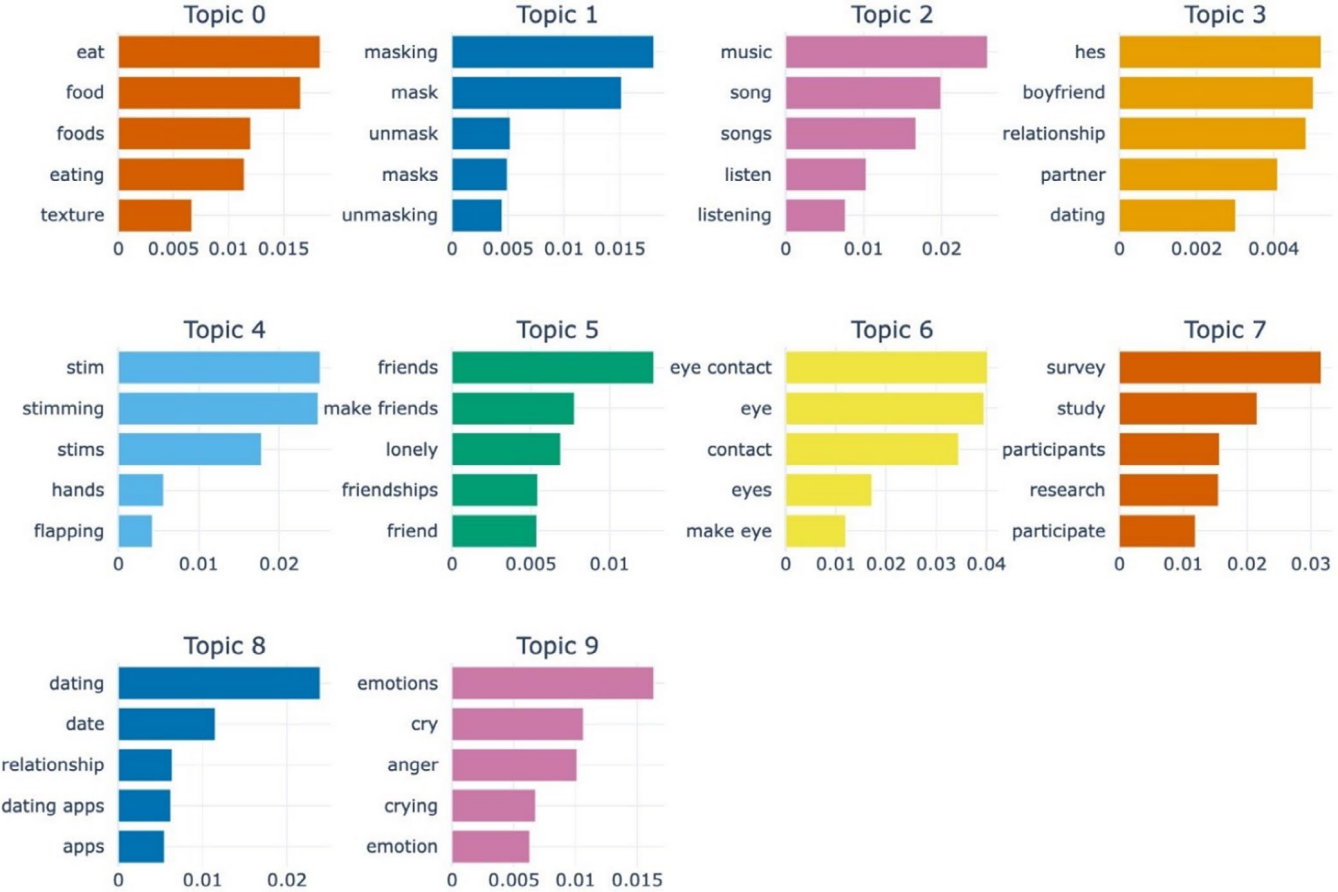
Top 5 representative words based on class‐based term frequency‐inverse document (c‐TF‐IDF) scores for the top 10 topics generated from posts from the subreddit *r/autism* (*n* = 174,102). The higher the c‐TF‐IDF score, the more relevant the word is in the context of a given topic. The ChatGPT‐4o generated labels for each of the topics are as follows: Topic 0 (Safe Foods and Eating Challenges in Autism), Topic 1 (Masking in Autism), Topic 2 (Music Listening Habits and Challenges), Topic 3 (Challenges in Relationships Involving Neurodivergence), Topic 4 (Stimming Behaviors in Autism), Topic 5 (Challenges in Making and Maintaining Friendships), Topic 6 (Challenges with Eye Contact in Autism), Topic 7 (Recruitment for Autism Research Surveys), Topic 8 (Dating and Relationships on the Autism Spectrum), Topic 9 (Understanding and Expressing Emotions in Autism).

Figure 1 depicts the top 10 most representative topics along with the top five keywords with the highest c‐TF‐IDF scores. These topics and keywords emerged from training a BERTopic model on the dataset containing posts exclusively from *r/autism*. The c‐TF‐IDF score measures the significance of a word within a specific topic in relation to other words. For example, the representation of topic 1 “Masking in Autism” is highly dependent on the word “masking” occurring in a post (Yao et al. 2023).

Table 1 is an overview of the top 10 discussion topics that emerged, as well as the respective topic labels generated by ChatGPT‐4o, top 10 representative keywords with corresponding c‐TF‐IDF scores, and the number of posts per topic.

**TABLE 1:** Top 10 topics that emerged with respective ChatGPT‐4o‐generated topic labels, top 10 keywords, and number of posts for the dataset containing posts from *r/autism*.

Topic number	Topic label generated by ChatGPT‐4o	Representative words (c‐TF‐IDF score)	Number of representative posts in topic
0	Safe foods and eating challenges in autism	“eat” (0.0183), “food” (0.0165), “foods” (0.0120), “eating” (0.0114), “texture” (0.0067), “taste” (0.0053), “meal” (0.0052), “cheese” (0.0049), “diet” (0.0049), “safe” (0.0048)	3247
1	Masking in autism	“masking” (0.0180), “mask” (0.0151), “unmask” (0.0052), “masks” (0.0049), “unmasking” (0.0045), “stop masking” (0.0026), “masked” (0.0022), “face” (0.0022), “ive” (0.0020), “people” (0.0020)	2029
2	Music listening habits and challenges	“music” (0.0259), “song” (0.0199), “songs” (0.0167), “listen” (0.0103), “listening” (0.0076), “lyrics” (0.0069), “playlist” (0.0054), “metal” (0.0046), “album” (0.0038), “listen music” (0.0037)	1946
3	Challenges in relationships involving neurodivergence	“hes” (0.0052), “boyfriend” (0.0050), “relationship” (0.0048), “partner” (0.0041), “dating” (0.0030), “together” (0.0029), “guy” (0.0024), “doesnt” (0.0024), “love” (0.0021), “said” (0.0021)	1471
4	Stimming behaviors in autism	“stim” (0.0251), “stimming” (0.0248), “stims” (0.0178), “hands” (0.0056), “flapping” (0.0042), “stop” (0.0039), “fingers” (0.0038), “hand” (0.0032), “rocking” (0.0031), “stim im” (0.0028)	1242
5	Challenges in making and maintaining friendships	“friends” (0.0128), “make friends” (0.0078), “lonely” (0.0069), “friendships” (0.0054), “friend” (0.0054), “friendship” (0.0045), “making friends” (0.0042), “alone” (0.0036), “hang” (0.0035), “make” (0.0034)	1047
6	Challenges with eye contact in autism	“eye contact” (0.0402), “eye” (0.0395), “contact” (0.0344), “eyes” (0.0172), “make eye” (0.0119), “look” (0.0107), “making eye” (0.0088), “staring” (0.0071), “stare” (0.0063), “looking” (0.0057)	873
7	Recruitment for autism research surveys	“survey” (0.0316), “study” (0.0216), “participants” (0.0157), “research” (0.0155), “participate” (0.0119), “link” (0.0106), “university” (0.0087), “participation” (0.0084), “please” (0.0074), “complete” (0.0074)	857
8	Dating and relationships on the autism spectrum	“dating” (0.0239), “date” (0.0115), “relationship” (0.0064), “dating apps” (0.0062), “apps” (0.0055), “relationships” (0.0046), “women” (0.0045), “men” (0.0038), “romantic” (0.0036), “dates” (0.0034)	840
9	Understanding and expressing emotions in autism	“emotions” (0.0163), “cry” (0.0106), “anger” (0.0101), “crying” (0.0068), “emotion” (0.0063), “angry” (0.0055), “emotional” (0.0047), “sad” (0.0045), “feeling” (0.0042), “feelings” (0.0042)	801

BERTopic identified 106,005 posts as outliers from the total of 174,102 posts in the dataset. This means that the outliers were not assigned to any specific topics. With the current hyperparameters, a total of 630 topics were generated. A high number of outliers is expected behavior in BERTopic as the model is designed to avoid forcing semantically incoherent posts into topics (Grootendorst, n.d.‐b). The following summary will focus on the top 10 most frequent topics that emerged.

The most frequently discussed topic (topic 0) was labeled as “Safe Foods and Eating Challenges in Autism”. It contained 3247 posts. This topic was characterized by discussions focusing on various aspects of food, meals, and eating behaviors. Key terms such as “eat”, “food”, “foods”, “eating”, and “texture” were prominent, reflecting users' conversations around meal choices, food texture, and taste.

The next most frequently discussed topic pertained to “Masking in Autism” (topic 1) and included 2029 posts. This topic was characterized by conversations related to the act of masking and unmasking. Common terms such as “masking”, “mask”, “unmask”, and “masks” appeared frequently.

“Music Listening Habits and Challenges” was the third most discussed topic (topic 2) with 1471 posts. Discussions centered around music, with key terms such as “music”, “song”, “songs”, and “listen”. Other keywords like “lyrics”, “metal”, and “album” suggest discussions about specific music genres and albums.

The fourth most frequently discussed topic (topic 3) revolved around “Challenges in Relationships Involving Neurodivergence”. This topic focused on discussions about relationships, with key terms like “love”, “boyfriend”, “relationship”, and “partner” reflecting conversations pertaining to various aspects of dating and relationship dynamics. Topic 3 consisted of 1471 posts.

“Stimming Behaviors in Autism” (topic 4) was the fifth most common topic of discussion and was represented by 1242 posts. This topic centered on discussions about stimming behaviors, with key terms like “stim”, “stimming”, and “stims”. Conversations about this topic were also characterized by specific stimming behavior keywords such as “flapping”, “rocking”, and movements involving “hands” and “fingers”.

The sixth most common topic was labeled as “Challenges in Making and Maintaining Friendships” with 1047 posts. This topic centered around users' experiences in forming friendships, with representative keywords such as “friends”, “make friends”, and “lonely”.

The seventh most discussed topic pertained to “Challenges with Eye Contact in Autism” and consisted of 873 posts. This topic focused on the significance and challenges of making eye contact in social situations. It was characterized by key terms such as “eye contact”, “eye”, and “contact”.

“Recruitment for Autism Research Surveys” was the eighth topic of discussion with 857 posts. This topic centered on efforts to recruit participants for various research studies, with keywords including “survey”, “study”, “participants”, “research”, “participate”, and “link”.

The ninth most discussed topic, “Dating and Relationships on the Autism Spectrum” (topic 8), included 840 posts. This topic focused on various aspects of dating, with conversations defined by key terms such as “dating”, “date”, and “dating apps”.

Lastly, “Understanding and Expressing Emotions in Autism” was the tenth most discussed topic from the dataset consisting of posts from the subreddit r/autism. This topic contained 801 posts centering on various aspects of emotions as representative keywords included “emotions”, “cry”, “anger”, and “feelings”.

### 3.2 Main Topics of Discussion in the 15 Autism‐Related Subreddits

This subsection summarizes the results of the topic modeling analysis conducted on posts from 15 autism‐related subreddits, excluding *r/autism*. The 15 subreddits included: *r/AutismInWomen, r/AutismTranslated, r/Autism_Parenting, r/AutisticAdults, r/AutisticCreatives, r/AutisticPeeps, r/AutisticPride, r/AutisticWithADHD, r/Autistic, r/SpicyAutism, r/autismmemes, r/evilautism, r/aspergers, r/aspergirls*, and *r/aspergers_dating*.

**FIGURE 2:**
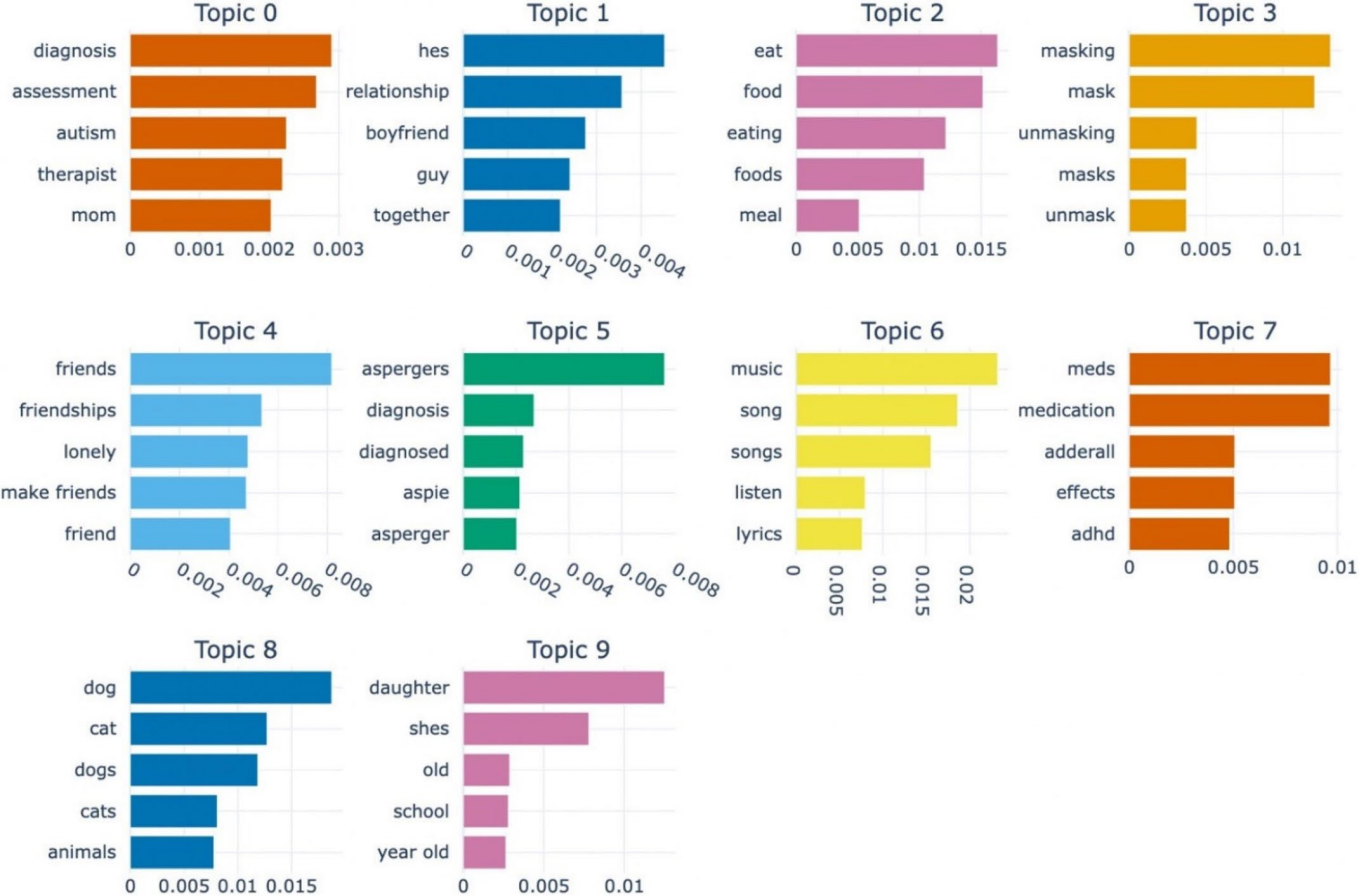
Top 5 representative words based on class‐based term frequency‐inverse document (c‐TF‐IDF) scores for the top 10 topics generated from posts from the 15 autism‐related subreddits (excluding *r/autism*). The higher the c‐TF‐IDF score, the more relevant the word is in the context of a given topic. The ChatGPT‐4o generated labels for each of the topics are as follows: Topic 0 (Navigating the Challenges of an Autism Diagnosis and Assessment), Topic 1 (Navigating Trust and Communication Challenges in Relationships with Partners on the Autism Spectrum), Topic 2 (Challenges with Eating and Food Sensory Issues), Topic 3 (Understanding and Managing Masking in Social Situations), Topic 4 (Struggles with Making Friends and Overcoming Loneliness), Topic 5 (Exploring Asperger's Syndrome Diagnosis and Personal Experiences), Topic 6 (Unique Experiences and Emotional Responses to Listening to Music), Topic 7 (Experiences with Medications), Topic 8 (Emotional Connections with Pets and Animal Care), Topic 9 (Navigating Challenges and Support for Children with Autism).

Figure 2 illustrates the resulting top 10 discussion topics along with their top 5 keywords after training a BERTopic model on the dataset of posts from the aforementioned 15 autism‐related subreddits (Table 2).

**TABLE 2:** Top 10 topics that emerged with respective ChatGPT‐4o‐generated topic labels, top 10 keywords, and number of posts for the dataset containing posts from 15 autism‐related subreddits (excluding *r/autism*).

Topic number	Topic label generated by ChatGPT‐4o	Representative words (c‐TF‐IDF score)	Number of representative posts in topic
0	Navigating the challenges of an autism diagnosis and assessment	“diagnosis” (0.0029), “assessment” (0.0027), “autism” (0.0022), “therapist” (0.0022), “mom” (0.0020), “appointment” (0.0019), “autistic” (0.0019), “diagnosed” (0.0017), “told” (0.0017), “said” (0.0016)	12,513
1	Navigating trust and communication challenges in relationships with partners on the autism spectrum	“hes” (0.0045), “relationship” (0.0036), “boyfriend” (0.0028), “guy” (0.0024), “together” (0.0022), “dating” (0.0021), “said” (0.0020), “doesnt” (0.0020), “told” (0.0018), “partner” (0.0018)	4029
2	Challenges with eating and food sensory issues	“eat” (0.01631), “food” (0.0151), “eating” (0.0121), “foods” (0.0104), “meal” (0.0051), “texture” (0.0047), “diet” (0.0047), “taste” (0.0045), “hungry” (0.0041), “meals” (0.0041)	3724
3	Understanding and managing masking in social situations	“masking” (0.0131), “mask” (0.0121), “unmasking” (0.0044), “masks” (0.0037), “unmask” (0.0037), “masked” (0.0021), “wearing” (0.0017), “unmasked” (0.0017), “face” (0.0016), “people” (0.0016)	3388
4	Struggles with making friends and overcoming loneliness	“friends” (0.0082), “friendships” (0.0053), “lonely” (0.0048), “make friends” (0.0047), “friend” (0.0041), “friendship” (0.0037), “alone” (0.0035), “loneliness” (0.0030), “close” (0.0025), “people” (0.0024)	3303
5	Exploring asperger's syndrome diagnosis and personal experiences	“aspergers” (0.0076), “diagnosis” (0.0027), “diagnosed” (0.0023), “aspie” (0.0021), “asperger” (0.0020), “symptoms” (0.0019), “social” (0.0018), “people aspergers” (0.0017), “diagnosed aspergers” (0.0017), “syndrome” (0.0016)	2876
6	Unique experiences and emotional responses to listening to music	“music” (0.0232), “song” (0.0186), “songs” (0.0155), “listen” (0.0079), “lyrics” (0.0076), “listening” (0.0061), “playlist” (0.0044), “band” (0.0035), “album” (0.0031), “listen music” (0.0030)	2722
7	Experiences with medications	“meds” (0.0097), “medication” (0.0096), “adderall” (0.0051), “effects” (0.0051), “adhd” (0.0048), “side effects” (0.0045), “medications” (0.0041), “dose” (0.0041), “taking” (0.0041), “prescribed” (0.0039)	2183
8	Emotional connections with pets and animal care	“dog” (0.0188), “cat” (0.0127), “dogs” (0.0119), “cats” (0.0081), “animals” (0.0078), “pet” (0.0060), “pets” (0.0057), “service dog” (0.0041), “animal” (0.0040), “vet” (0.0035)	1636
9	Navigating challenges and support for children with autism	“daughter” (0.0125), “shes” (0.0078), “old” (0.0029), “school” (0.0028), “year old” (0.0026), “old daughter” (0.0026), “kids” (0.0025), “doesnt” (0.0022), “year” (0.0022), “speech” (0.0021)	1441

From the dataset containing posts from the 15 autism‐related subreddits, BERTopic classified 170,882 posts as outliers from the total 291,756 posts.

Outliers were not grouped into any specific topics. Under the current hyperparameters, 806 topics were generated in total. The summary below provides an overview of the top 10 most frequent topics.

The most frequent topic from this dataset had the label “Navigating the Challenges of an Autism Diagnosis and Assessment” (topic 0). It comprised 12,513 posts discussing the experiences related to the diagnosis and assessment of autism. This topic was characterized by keywords such as “diagnosis”, “assessment”, “appointments”, and “therapist”.

The second most frequent topic, “Navigating Trust and Communication” Challenges in Relationships with Partners on the Autism Spectrum” (Topic 1) had 4029 posts. Discussions frequently centered on experiences with boyfriends and partners as key terms such as “relationship”, “boyfriend”, and “guy” were commonly included in the related discussions.

“Challenges with Eating and Food Sensory Issues” (Topic 2) was the third most discussed topic and consisted of 3724 posts. Posts under this topic included users sharing their experiences regarding eating, preferences for specific textures, and difficulties with meal selections. Keywords such as “eat”, “food”, “eating”, “meal”, “texture”, and “diet” frequently appeared in these discussions.

The fourth topic of discussion was labeled “Understanding and Managing Masking in Social Situations” (Topic 3). This topic had 3388 posts that encompassed user experiences with masking and unmasking. Representative keywords included “masking”, “unmasking”, “masks”, “masked”, and “wearing”.

“Struggles with Making Friends and Overcoming Loneliness” was the fifth most commonly discussed topic. It featured 3303 posts that explored the challenges and experiences related to forming and maintaining friendships. Frequently occurring keywords in these posts included “friends”, “friendships”, “lonely”, and “make friends”.

The sixth topic of discussion, “Exploring Asperger's Syndrome Diagnosis and Personal Experiences” had 2876 posts. Representative keywords such as “aspergers”, “diagnosis”, “symptoms”, and “social” highlighted the focus on understanding and navigating an Asperger's diagnosis.

“Unique Experiences and Emotional Responses to Listening to Music” (Topic 6) emerged as the seventh most commonly discussed topic. It included 2722 posts centered on the enjoyment and exploration of music with keywords such as “music”, “song”, “listen”, and “lyrics” frequently occurring frequently in the posts.

The topic “Experiences with Medications” was the eighth topic of discussion with 2183 posts. Posts classified under this topic focused on experiences and practical aspects of medication management. Some representative keywords included “meds”, “medication”, “adderall”, and “side effects”.

The ninth topic of discussion was labeled as “Emotional Connections with Pets and Animal Care”. The 1636 posts on this topic were characterized by keywords such as “dog”, “cat”, “pets”, “animals”, and “service dog”.

“Navigating Challenges and Support for Children with Autism” was the tenth most common topic of discussion for this dataset of posts from the 15 autism‐related subreddits. It comprised 1441 posts focused on the experiences of parents, with posts highlighted by keywords such as “daughter”, “school”, and “speech”.

## 4 Discussion

Social media platforms play a crucial role in shaping public perceptions of neurodevelopmental conditions, such as autism, by providing spaces for community interaction and content sharing. While often fostering connections and support for autistic individuals, social media platforms also serve as valuable sources for understanding personal experiences and diverse perspectives surrounding these conditions. As in previous works (Fong et al. 2024a, 2024b), this study implemented a state‐of‐the‐art NLP topic modeling method (i.e., BERTopic [Grootendorst 2022]) to identify the main topics of discussion across 16 autism‐related Reddit subreddits. Two separate analyses were conducted: (i) an analysis of 174,102 posts from the main *r/autism* subreddit with posts spanning from 2009 to 2023, and (ii) an analysis of 291,756 posts from 15 autism‐related subreddits (i.e., *r/AutismInWomen, r/AutismTranslated, r/Autism_Parenting, r/AutisticAdults, r/AutisticCreatives, r/AutisticPeeps, r/AutisticPride, r/AutisticWithADHD, r/Autistic, r/SpicyAutism, r/autismmemes, r/evilautism, r/aspergers, r/aspergirls, r/aspergers_dating*), with posts dated from 2010 to 2023. ChatGPT‐4o was used to generate descriptive labels for the extracted topics according to their respective representative keywords and posts.

Results of the present work show that discourse in *r/autism* (i.e., the first analysis) comprises a broad range of topics, including behaviors (e.g., stimming, maintaining eye contact, expressing emotion), sensory issues, and concerns about social relationships (e.g., platonic, romantic). Similar topics were explored within the other 15 autism‐related subreddits (i.e., the second analysis). However, posts from this second subset of data also covered specific emotional experiences and practical concerns, such as managing a diagnosis, experiences with medication, and the day‐to‐day aspects of living with autism and supporting others.

The following discussion of the overall results is organized into subsections reflecting key themes often associated with autism: (i) challenges in social communication and interaction across various contexts, and (ii) patterns of behavior, interests, or activities that may appear repetitive or highly focused. An additional miscellaneous subsection is included to address topics that do not fall within these categories. Any descriptions of posts are based on the extracted representative posts for each topic identified by BERTopic.

### 4.1 Social Communication and Social Interactions Across Multiple Contexts

The findings from the analyses highlight various experiences and content related to differences in social communication and interaction across multiple contexts, as described in discussions about autism. These differences, often framed by autistic individuals as arising from divergent social norms rather than deficits, emerge at multiple levels, including navigating trust and communication issues in romantic relationships, challenges in forming and maintaining friendships, and discomfort with eye contact or processing emotions. In the context of romantic relationships, users on Reddit sought insight into understanding their autistic partner's behaviors and how to offer appropriate support, while others looked for advice on how to date while on the spectrum. For some autistic individuals, having a partner might be a way to navigate and manage their own difficulties, offering a sense of support and understanding that eases the challenges associated with social interactions (Yew et al. 2021). In examining the Reddit posts, a clear distinction emerges between two different topics in *r/autism*: Topic 3, which addresses the experiences of individuals in ongoing, long‐term romantic relationships, and Topic 8, which focuses on the early stages of dating. Topic 3 focuses on individuals reflecting on their experiences in long‐term romantic relationships, often involving partners on the autism spectrum. Posts in this topic address challenges such as communication difficulties, emotional understanding, and the impact of autism on relationship dynamics. Users share frustrations with the lack of awareness about autism in their relationships and the emotional strain that can result. In contrast, Topic 8 deals with dating and the early stages of romantic relationships. Here, users discuss challenges like navigating dating apps, disclosing their autism diagnosis to potential partners, and managing social anxiety during the early stages of dating. While Topic 3 centers on long‐term relational dynamics, Topic 8 is focused on initial interactions and the complexities of starting romantic relationships as an autistic individual. Additionally, the emerged topics highlight the difficulties that autistic individuals face in forming and maintaining friendships, as well as dealing with feelings of isolation due to these challenges. Some users discussed conflicting feelings of wanting friends but also not wanting friends, as social interactions can be taxing or stressful. Others sought tips on forming friendships, with one user sharing how joining a common interest group positively impacted their social life.

Building on these challenges, three specific topics further illustrate the complexity of social communication issues for autistic individuals. First, the discomfort many autistic individuals experience with eye contact, which is critical for social interaction, was a key focus of the discussions. Users discussed the challenges associated with making eye contact, with some finding it physically uncomfortable, while others expressed confusion or internal conflict due to years of masking. They shared experiences of briefly holding eye contact or struggling with whether to maintain it, seeking advice on whether to continue attempting or abandon it altogether. Second, users address the pressure to hide or mask autistic traits in public. Many users described masking as a form of self‐regulation and an attempt to navigate societal expectations, even though it often came with confusion in distinguishing their authentic selves from their masked behaviors. Over time, masking becomes automatic for many, but it takes a significant emotional toll, leading to burnout or emotional crashes. Users sought advice on how to take breaks from masking or stop altogether while managing the social and personal consequences. Third, discussion topics highlight the difficulties autistic individuals face in recognizing, expressing, and processing emotions. Users described a disconnect between feeling emotions in the moment and processing them later, with some questioning whether their emotional responses were genuine or forced. These behaviors and strategies, including masking and seeking connections through relationships or friendships, are often discussed by autistic individuals as ways to cope with challenges and manage one's own difficulties in a neurotypical‐dominated society.

### 4.2 Patterns of Behavior, Interests, and Activities

In addition to the social communication challenges discussed in Section 4.1, several topics emerged regarding patterns of behavior, interests, or activities in autistic individuals. A recurring theme was eating behaviors, which were a major topic of discussion across both sets of analyses. These topics highlighted how sensory sensitivities, such as food texture and taste, contribute to eating habits. Users described their eating routines as driven by the need for predictability, safety, and control, which often leads to a limited diet. Many expressed anxiety around trying new foods or eating outside their comfort zones, with external pressures from family and social situations adding to their difficulties. Similarly, topics related to music listening habits and experiences further exemplify restricted and repetitive interests. Autistic individuals reported often engaging in repetitive listening behaviors, repeatedly playing specific songs or genres. Many shared experiences of focusing intensely on the music rather than the lyrics, struggling to transition between songs, and forming deep emotional connections to certain tracks, which could leave them feeling vulnerable when sharing their musical preferences with others who may not understand their significance.

Finally, users' posts also focused on the physical, repetitive actions that autistic individuals use to self‐regulate or express emotions. These behaviors, such as hand‐flapping or rocking, were discussed as both comforting and necessary for emotional regulation. Users reflected on the societal perceptions of stimming, especially those questioning their autism diagnosis, and described how these behaviors can lead to increased anxiety and the need for further stimming, thus perpetuating a cycle.

### 4.3 Miscellaneous Topics

Several other topics emerged from the analyses that provide valuable insights into the broader experiences of individuals within the autism community. In the topic “Navigating the Challenges of an Autism Diagnosis and Assessment”, users shared their personal experiences with diagnosis and assessment. Some discussed their feelings upon receiving an autism diagnosis, while others reflected on their struggles with social difficulties, seeking clarity on whether their experiences warranted an official diagnosis. Representative posts on this topic revolve around emotional suffering, childhood trauma, and struggles with identity, often in relation to self‐diagnosed autism. Many users describe experiences of familial abuse, loneliness, depression, and self‐destructive tendencies, alongside a deep yearning for connection and acceptance. A recurring theme is the difficulty of finding a place in the world while grappling with the awareness of not fitting into social or familial expectations. Psychological distress and the desire for support are central, yet the instability of personal circumstances and the lack of adequate resources often leave these struggles unresolved. Additionally, many users express uncertainty about self‐diagnosis, questioning whether their challenges stem from undiagnosed conditions or personal shortcomings. This lack of external validation reinforces a sense of isolation, making the search for answers even more daunting.

A similar pattern emerges in discussions about Asperger's syndrome, particularly among adults who have masked their traits for years. Many describe an identity crisis upon realizing they may be autistic, struggling to distinguish between their inherent personality and the effects of autism. Self‐doubt is common, especially when their experiences do not perfectly align with standard diagnostic criteria or when long‐standing coping mechanisms obscure the signs. Users frequently mention sensory sensitivities, social exhaustion, and emotional regulation difficulties, often recognizing similar traits in undiagnosed family members. The lack of research on female presentations of autism further complicates self‐identification, leading many to rely on personal blogs rather than clinical studies. Overall, these experiences highlight the profound impact of late self‐recognition and the challenges of navigating an autism diagnosis in adulthood.

Another topic, “Emotional Connections with Pets and Animal Care”, revolved around the importance of emotional regulation and supportive relationships, particularly with animals. Users shared how pets can provide emotional comfort and stability, especially when faced with social misunderstandings and emotional challenges. One user explored the metaphorical role of animal companionship in understanding and managing their emotions, highlighting how pets offer a nonjudgmental and calming presence, which is crucial for individuals who may struggle with social interactions.

The topic “Navigating Challenges and Support for Children with Autism” focused on the experiences of parents raising children with autism. These discussions centered on the emotional, social, and educational challenges parents face while advocating for their children's needs and seeking understanding and acceptance in various environments.

Lastly, the analysis revealed that Reddit also serves as a platform for recruiting participants for autism research. “Recruitment for Autism Research Surveys” focused on efforts to engage individuals in autism research studies.

## 5 Limitations

The present work has a number of limitations. Firstly, over 50% of the posts were categorized by BERTopic as outliers and therefore not assigned to a particular topic of discussion for both subsets of subreddit data analyzed. Future work could focus on further tuning model parameters to reduce outliers while maintaining low noise (i.e., avoiding misassigned outliers). Secondly, while BERTopic is a validated and state‐of‐the‐art approach for topic modeling, we did not conduct an independent evaluation of the model's performance, such as human annotation or confusion matrix analyses. This limitation reflects the study's focus on an unsupervised machine learning‐based identification of themes across a large dataset rather than validation against human‐coded ground truth. However, validating the model through comparison with human judgments represents an important direction for future research. Future research could also complement topic modeling results with additional validation methods to further assess the reliability and interpretability of the model's results. Thirdly, the comments written in response to the posts were not considered for the topic modeling analysis. Subthreads within the comments may offer a further understanding of autism‐related discussions on Reddit. Fourthly, the analysis could not distinguish between autistic and non‐autistic users. This limitation makes it difficult to identify whether the perspectives shared in posts represent firsthand experiences of autistic individuals or observations and questions from non‐autistic users. Fifthly, the present work only considers one social media platform (Reddit) and could benefit from including discussions from additional platforms to provide a more comprehensive understanding of online autism‐related discourse. Similarly, the data analyzed in this study were limited to the dataset used, which only included subreddits available within the publicly compiled dataset. As a result, other autism‐related subreddits may exist but were not accessible for analysis. Finally, while topic modeling provides valuable insights into recurring themes of discussion, it does not capture the nuanced emotional aspects of the discourse.

## 6 Conclusions

In conclusion, this study leveraged the unique insights provided by social media platforms to identify prevalent discussion topics within Reddit‐based autistic communities. Utilizing the BERTopic NLP topic modeling approach and following the methodology outlined in Fong et al. (2024a, 2024b), we analyzed discussions made between June 2005 to December 2023 across 16 autism‐related Reddit subreddits. The findings highlight the experiences surrounding social communication and behavioral patterns, giving insight into the emotional and practical concerns faced by autistic individuals. Key themes included navigating relationships and the challenges of social interactions, as well as discussions on stimming and sensory sensitivities. Moreover, the findings demonstrate that Reddit serves as an invaluable platform for sharing personal experiences, thereby contributing to a deeper understanding of the needs and perspectives of autistic individuals.

Findings also indicate unmet needs among Reddit users who self‐identify as autistic, particularly in areas such as diagnosis and assessment, relationships, and sensory issues. These insights can inform efforts to better support autistic individuals, aligning current practices with the priorities emerging from online discussions. Future research should examine how well existing clinical and support frameworks address these expressed needs to derive targeted clinical insights. However, it is important to acknowledge that Reddit users who self‐identify as autistic may not fully overlap with those meeting clinical criteria for autism as defined by the fifth edition of the *Diagnostic and Statistical Manual of Mental Disorders* (DSM‐5) (American Psychiatric Association 2013). Thus, while these findings offer valuable perspectives, they may not fully represent the broader autism spectrum. Additionally, since the analysis was limited to English‐language discussions, clinical insights may not be generalizable to non‐English‐speaking individuals or different geographical contexts.

Although many of the topics identified in our study align with priorities highlighted in prior research, such as experiences with diagnosis, masking, loneliness, relationships, and sensory issues, some themes emerging from our analysis are less frequently discussed in previous literature. Notably, “Safe Foods and Eating Challenges in Autism” was the most prominent topic within *r/autism*, and “Music Listening Habits and Challenges” was a top‐10 theme in both *r/autism* and across the 15 other autism‐related subreddits. These findings suggest that certain aspects of autistic experiences, particularly in relation to food and music, may be underexplored in existing research. Future studies should further investigate these topics to derive actionable clinical insights.

Future research could also explore how discussions evolve over time, particularly in response to changes in public awareness, policy, and social media dynamics. Additionally, investigating the perspectives of different demographic groups within autistic communities, such as gender‐diverse individuals or those from different cultural backgrounds, could offer further insights into how autism‐related discourse varies across subpopulations. Furthermore, expanding the analysis to incorporate sentiment analysis and other NLP techniques could provide deeper insights into the emotional tone and underlying attitudes expressed in online discussions, further enriching the interpretation of autism‐related discourse. Finally, future research could explore the use of topic models for real‐time classification of new posts, allowing for the continuous monitoring of evolving discussions and emerging themes in autism‐related online communities.

Importantly, the discussions that emerged from the current work also reflect a deeper, universal challenge: the effort to synchronize one's internal sensations and emotions with external social norms. For many autistic individuals, this balance often involves navigating complex processes, such as managing the desire for authenticity in contexts where societal expectations might require masking or adapting behaviors. This struggle to reconcile personal subjectivity with external norms highlights the broader, shared human experience of negotiating individual identity within societal frameworks, though it is especially pronounced in autistic experiences.

## Author Contributions

**Seraphina Fong, Alessandro Carollo, Gianluca Esposito:** conceptualization. **Seraphina Fong:** methodology, investigation, software. **Seraphina Fong, Alessandro Carollo:** formal analysis. **Seraphina Fong, Alessandro Carollo:** writing – original draft preparation. **Seraphina Fong, Alessandro Carollo, Giacomo Vivanti, Daniel S. Messinger, Dagmara Dimitriou, Gianluca Esposito:** review and editing. **Gianluca Esposito:** supervision. All authors have read and agreed to the published version of the manuscript.

## Conflicts of Interest

The authors declare no conflicts of interest.

## Supporting information


**Data S1.** Hyperparameters of the BERTopic model.

## Data Availability

The raw data used for the present study was obtained from a public repository accessed here in light of Reddit's application programming interface (API) privacy policy changes in June 2023: https://www.reddit.com/r/pushshift/comments/1akrhg3/separate_dump_files_for_the_top_40k_subreddits/. The repository is a large public dataset containing posts and comments from the top 40,000 subreddits obtained and stored publicly by an approved Reddit moderator.
