# Supplementary material for: Autism Spectrum Disorders Discourse on Social Media Platforms: A Topic Modeling Study of Reddit Posts
Source: Autism Res. 2025 Jun 5;18(8):1608–19. doi: 10.1002/aur.70066 (PMC12384743; doi:10.1002/aur.70066)
Supplement: Supplementary file 1 — Data S1. Hyperparameters of the BERTopic model. [file AUR-18-1608-s001.docx]

| **Module (purpose)** | **Hyperparameter** | **Value** |
| --- | --- | --- |
| Embedding model (convert Reddit posts into numerical representations i.e., embeddings) | English Sentence transformer model | all-MiniLM-L6-v2 |
| UMAP model (dimensionality reduction of extracted embeddings) | Number of neighbors | 15 |
|  | Number of components | 5 |
|  | Minimum distance | 0.1 |
|  | Random state | 42 |
| HDBSCAN model (cluster embeddings of similar posts) | Minimum cluster size | 15 |
| CountVectorizer (tokenize topics) | Stop words | NLTK english stop words + [‘http’, ‘https’, ‘amp’, ‘com’] |
|  | Ngram range | (1,2) |
| Class TF-IDF model (create topic representation of each cluster of similar posts) |  | Default parameters |

Table S1: Overview of hyperparameters set and descriptions of each BERTopic. Most default out-of-the-box values were retained.
